# Lessons Learned From Translational Research in Neuromuscular Diseases: Impact on Study Design, Outcome Measures and Managing Expectation

**DOI:** 10.3389/fgene.2021.759994

**Published:** 2021-12-07

**Authors:** Georgia Stimpson, Mary Chesshyre, Giovanni Baranello, Francesco Muntoni

**Affiliations:** ^1^ Developmental Neuroscience Research and Training Department, Dubowitz Neuromuscular Centre, Faculty of Population Health Sciences, UCL Great Ormond Street Institute of Child Health, University College London, London, United Kingdom; ^2^ NIHR Great Ormond Street Hospital Biomedical Research Centre, UCL Great Ormond Street Institute of Child Health, University College London, London, United Kingdom

**Keywords:** spinal muscular atrophy, duchenne and becker muscular dystrophy, translational research, trial design, inclusion criteria, matching criteria

## Abstract

Spinal Muscular Atrophy (SMA) and Duchenne Muscular Dystrophy (DMD), two of the most common, child onset, rare neuromuscular disorders, present a case study for the translation of preclinical research into clinical work. Over the past decade, well-designed clinical trials and innovative methods have led to the approval of several novel therapies for SMA and DMD, with many more in the pipeline. This review discusses several features that must be considered during trial design for neuromuscular diseases, as well as other rare diseases, to maximise the possibility of trial success using historic examples. These features include well-defined inclusion criteria, matching criteria, alternatives to placebo-controlled trials and the selection of trial endpoints. These features will be particularly important in the coming years as the investigation into innovative therapy approaches for neuromuscular diseases continues.

## Introduction

Translational research in neuromuscular diseases has evolved dramatically over the past few years, with dozens of clinical trials in the pipeline for several conditions, and a few approved novel treatments that are now available to patients in clinic. This rapid expansion of translational research and therapeutics has not been devoid of pitfalls and disappointments; however, these missteps have led to customized trial outcome measures and improved clinical trial design for neuromuscular diseases. The lessons learned from clinical trials, and the wider implementation of approved treatments in the real world, are ultimately moving the neuromuscular field towards individualised treatments based on patient characteristics. Two neuromuscular diseases, Spinal Muscular Atrophy (SMA) and Duchenne Muscular Dystrophy (DMD) present case studies of the massive work done over the past two decades that has translated preclinical into clinical research. This work has included developing new disease-specific outcome measures, well-designed clinical trials, and identifying the optimal patient population to be investigated in the clinical trials.

### Background

SMA is an autosomal recessive condition with an incidence of 1 in 6,000 to 20,000 live births (Jones et al., 2015). SMA is caused by low levels of survival motor neuron (SMN) protein, resulting from mutations in the *SMN1* gene. The paralog *SMN2* gene is only able to make markedly reduced functional SMN protein, approximately 10% of the levels produced by *SMN1*, due to skipping of exon 7, leading to an ineffective protein. In SMA, muscle weakness and atrophy predominantly affect the proximal lower limbs, followed by a progressive decline of strength in the upper limbs and axial muscles (Wadman et al., 2018). SMA types are defined by age at symptom onset and the maximum motor milestone achieved. Those with SMA I have symptom onset before 6 months and never sit independently, in fact very few achieve motor milestones such as head control or rolling (Bruggen, 2016; de Sanctis et al., 2016). Those with SMA II have symptom onset between 7 and 18 months, and can sit independently but never walk unaided, while those with SMA III have symptom onset after 18 months and can walk independently, although they can lose this ability with time. SMA IV has onset during adulthood (Mercuri et al., 2012). In general, individuals with SMA II/III may experience an improvement in motor function in the first few years of life, until the ages of five and six to seven respectively (Wadman et al., 2018; Coratt et al., 2020; Coratti et al., 2020); this is usually followed by a fairly stable period and then a non-linear decline with time (Mercuri et al., 2016) which can be difficult to capture using some of the motor function assessment methods (Kaufmann et al., 2011; LoMauro et al., 2016).

DMD is an X-linked disease affecting approximately 1 in 5,000 male live births (Ryder et al., 2017). DMD is a progressive disorder characterised by muscle wasting and weakness. The eight reading frame rule states that DMD mutations that disrupt the reading frame and lead to loss of dystrophin protein are associated with DMD, whilst in-frame DMD mutations that lead to a reduction in the amount or size of dystrophin protein are associated with the milder Becker muscular dystrophy (Bladen et al., 2015). The reading frame rule has been reported to hold true for DMD in 93% in the Treat NMD global database, 91% in the Leiden DMD database and 96% in the French UMD DMD database (Aartsma-Rus et al., 2006; Tuffery-Giraud et al., 2009; Bladen et al., 2015). However, in the Leiden DMD database, Aartsma-Rus et al. (2006) observed that when DMD mutations were confirmed at the RNA level, rather than just the DNA level, over 99.5% fit with the reading-frame rule (Aartsma-Rus et al., 2006). Notable exceptions include large in-frame deletions and duplications, or even small in-frame deletions or other mutations affecting critical domains of dystrophin, such as the dystroglycan binding domain.

Boys with DMD are typically diagnosed between the age of 3–5 years (Bushby et al., 1999; Mohamed et al., 2000; Ciafaloni et al., 2009; van Ruiten et al., 2014) and experience a continuous decline in motor function after approximately age 7 (Mazzone et al., 2011; Ricotti et al., 2016). In glucocorticoid (GC) naive boys, ambulation is typically lost before the age of 12. The chronic use of GCs (Prednisone/Prednisolone or Deflazacort) has been shown to prolong ambulation up to the age of 12–15 years (Ricotti et al., 2013; Sussman et al., 2020; Zhang et al., 2021). In addition to progressive muscle weakness in the lower limbs leading to loss of ambulation, DMD boys also show progressive weakness of the upper limbs, respiratory and cardiac muscles, which leads to cardiomyopathy and respiratory insufficiency in later life (Spurney, 2011; Ricotti et al., 2019; Bello et al., 2020). Patients with DMD have a reduced life expectancy, with a mean age of survival in the late 20s (Eagle et al., 2002; Eagle et al., 2007; Passamano et al., 2012). Both Ricotti et al. (2016) and Darmahkasih et al. (2020) have noted high rates of comorbidities of neurodevelopmental, behavioural and emotional symptoms, including intellectual disabilities, anxiety and inattention.

The treatment landscapes for SMA and DMD have evolved dramatically in recent years. Three disease-modifying treatments have been authorised by the US Food and Drug Administration (FDA) and European Medicines Agency (EMA) for SMA, Onasemnogene abeparvovec (Zolgensma^TM^), Risdiplam (Evrysdi^TM^) and Nusinersen (Spinraza^TM^). Onasemnogene abeparvovec is a one-time genetic replacement adeno associated virus (AAV) therapy that contains a functional *SMN1* gene. Nusinersen and Risdiplam both act on the splicing of the *SMN2* gene and induce the retention of exon 7 in the transcript, hence allowing the increase in the production of functional SMN. Risdiplam is a daily oral medication, whereas Nusinersen is administered intrathecally via lumbar puncture, three times in a year after the loading phase in the few months that requires more frequent administration.

While GCs remain the mainstay of pharmacological treatment for DMD, several DMD mutation-specific treatments have been developed. One of these, Ataluren, enables ribosomal readthrough of premature stop codons present in 10–15% of patients with nonsense mutations (Bushby et al., 2014). It has been approved by EMA for use in the EU (Committee for Medicinal Products for Human Use (CHMP), 2018a). In separate efforts, several groups have developed exon skipping therapies, which aim to restore the *DMD* reading frame isoforms in patients with out-of-frame deletions and to allow the expression of partially functional dystrophin (Niks and Aartsma-Rus, 2017). Several such antisense oligonucleotide (ASO) drugs, which require weekly intravenous administration, have been developed in the last decade, targeting exon 51 [Eteplirsen (Mendell et al., 2013; Mendell et al., 2016)], exon 53 [Golodirsen (Frank et al., 2020) and Viltolarsen (Clemens et al., 2020)] and exon 45 [Casimersen (Wagner et al., 2021)]. 14, 10, and 9% of all DMD boys are amenable to skipping of exons 51, 53, and 45, respectively (Bladen et al., 2015). All four of these drugs are approved in the US (Shirley, 2021; Heo, 2020; Syed, 2016; U.S. Food and Drug Administration, 2020) and Viltolarsen also in Japan (Dhillon, 2020), but they have not been approved yet in Europe. This is due to differences in the emphasis given to surrogate outcome measures (such as dystrophin restoration in muscle) between the different regulatory agencies.

## Impact on Study Design

### Inclusion Criteria and Participants Selection

Despite the exponential increase of clinical trials in SMA and DMD, both conditions present significant statistical challenges, due to the systemic nature of the disease and the heterogeneity in clinical manifestations. For this reason, significant work has been done to optimise clinical trial design, and several lessons can be learnt from these previous experiences. When designing clinical trials in neuromuscular diseases (and other rare diseases), there is a trade-off between enrolling the subpopulations who are most likely to benefit from the treatment (the “optimal” population) and having less strict inclusion criteria to enrol a representative sample of the overall patient population. This is important to allow a broad label for the medicinal product so that not only those patients within the narrow margin of the trial inclusion criteria could benefit from them. Historically, the field has also faced several trials which were unsuccessful due to insufficient treatment effects, such as Phenylbutyrate (Mercuri et al., 2007), Valproic Acid (Swoboda et al., 2010), Raxone (Servais et al., 2020) and more recently the anti-myostatin trials (ClinicalTrials.gov: NCT03039686 and NCT02310763). For an overview of key features for all of the trials referenced in this review refer to Table 1.

**TABLE 1:** Overview of key trials in DMD and SMA discussed in this review.

Trial Number (Name)	Trial drug	Biology	Trial stage	Trial population	Trial features	Primary endpoint	Maximum follow up	N	Complete (mm/yy)
DMD									
NCT03769116	SRP-9001	AAV mediated gene therapy with micro-dystrophin	2	• 4–7 years, • Stable GCs for 12 weeks prior	Multicentre, randomised, placebo-controlled cross-over trial for efficacy	Change in Micro-Dystrophin protein expression (western blot) Change in NSAA	48 weeks	41	No
NCT04281485	PF-06939926	AAV mediated gene therapy with mini dystrophin	3	• 4–7 years old,• Ambulant, • Stable GCs for 3 months prior	Randomised (2:1), double-blind, placebo-controlled efficacy trial	NSAA change from baseline	52 weeks	∼99	No
NCT02310763	Domagrozumab (PF-06252616)	Anti-myostatin adnectin	2	• 6–16 years, • 4SC ≤ 6.4 s, ≥1.32 s, • GC use for >6 months prior, stable GCs for 3 months prior	Randomized, double-blind, placebo-controlled, multi-dose, efficacy and safety study	Adverse events Change in 4SC	49 weeks	121	Yes (08/18)
NCT03039686	RG6206 (RO7239361)		2/3	• 6–11 years, • Ambulant (no assistance), • NSAA≥ 15 at baseline• 4SC ≤8 s, • GC use	Randomized, double-blind, placebo-controlled, multi-dose, efficacy and safety study	Change in NSAA score	48 weeks	166	Yes (12/20)
NCT02255552 (PROMOVI)	Eteplirsen	PMO for exon 51 skipping	3	• 7–16 years, • Stable GCs for 24 weeks prior, • 6 MWD ≥300 m (main analysis 300–450 m),• Stable pulmonary and cardiac function	Open-label, multi-centre, study with concurrent control arm (not treated, not amenable) for efficacy and safety	Change in 6 MWD	96 weeks	109	Yes (01/21)
NCT03907072	WVE-210201 (Suvodirsen)		2/3	• 5–12 years, • Ambulant, • Stable pulmonary and cardiac function,• Amenable to exon 51 skipping,• GC use for >6 months prior, stable GCs for 3 months prior	Randomized, double-blind, placebo-controlled, multi-dose, efficacy and safety study with open-label extension	Change in Dystrophin Level and NSAA score	48 weeks	6	Yes (05/21)
NCT01826487 (ACT-DMD)	Ataluren	Small molecule that restores dystrophin synthesis by allowing ribosomes to read through premature stop codons	3	• 7–16 years, • Nonsense mutation DMD, • GC use for >6 months prior, stable GCs for 3 months prior, • 6 MWD ≥150 m, 6 MWD ≤80% predicted for age	Efficacy and Safety Study placebo controlled	6 MWD	48 weeks	230	Yes (08/20)
SMA									
NCT02292537 (CHERISH)	Nusinersen (ISIS 396443)	SMN2-directed RNA splicing modifier	3	• 2–12 years, • Onset ≥6 months old, • HFMSE ≥ 10 and ≤ 54 at baseline, • Independent sitter,• Never ambulant	Randomised, double-blind, sham-procedure controlled study to assess efficacy and safety	Change in HFMSE score	15 months	126	Yes (02/21)

This has highlighted the need to specify inclusion/exclusion criteria that will lead to the enrolment of a patient population with better defined natural history data or a higher likelihood of response to treatment. A case study for this was the experience in the Nusinersen sham-controlled trials for SMA. In the CHERISH study in patients with SMA II (Mercuri et al., 2018), strict inclusion/exclusion criteria were applied, including a relatively high functional motor score on the Hammersmith Functional Motor Scale Expanded (HFMSE) scale, between 10 and 54, a maximum age at enrolment of 12 years and the absence of contractures and severe scoliosis. These well-defined criteria were crucial to the success of the study, which ultimately led to the approval of Nusinersen.

The issue of identifying well-defined inclusion criteria, which take into account functional variability and different predicted long-term trajectories, has become obvious in recent years for DMD. Almost invariably all recent trials in DMD only include patients receiving standard of care (SoC) treatments, such as those being on a stable dose and regime of GCs (see ClinicalTrials.gov: NCT02255552, NCT01826487 and NCT04281485 for example). This can lead to potential bias in patient selection, particularly in younger boys. In DMD, disease severity has been correlated with GC starting age, with patients with more severe phenotypes more likely to start GCs at a younger age (Ricotti et al., 2016). Similarly, Muntoni, et al. (2019) used latent class trajectory analysis to cluster DMD boys into four groups based on the North Star Ambulatory Assessment (NSAA) score. They found that those in the two clusters with fastest disease progression (class 1 and class 2) had an earlier mean age of starting GCs than those in the two clusters with slower disease progression. This suggests that by preferentially offering GCs to young DMD boys with early clinical presentation there is a risk of bias towards selecting those on a more severe trajectory. Removing these criteria means a more representative sample of DMD patients, although it leads to trajectory heterogeneity which represents an added difficulty for disease progression modelling.

To avoid these complexities, a broader population of patients, with a well-defined subpopulation with stricter clinical and functional inclusion/exclusion criteria has been shown in the literature as a viable approach. In the ACT DMD trial (McDonald et al., 2017) for Ataluren, a sub-population of interest, those boys whose 6 Minute Walking Distance (6 MWD) was between 300 and 400 m, was pre-specified, but there was also a broader enrolment. The argument for this specification was that patients who have better functional abilities (>400 m 6 MWD) remain stable over the 48 weeks of the study. Therefore, as Ataluren aims to induce stability, this population were not optimal for demonstrating treatment effect. On the other hand, it was argued that the weakest patients (6 MWD <300 m) had a high rate of interpatient variability, often having started the very rapid phase of disease progression leading to loss ambulation within a short period of time. This variability and different stages of the disease can cloud the ability to demonstrate a treatment effect. By pre-specifying a sub-population of interest, but also having broader inclusion criteria, the researchers maximised the chance of getting clear estimates of treatment effect, whilst also beginning to understand the variation in treatment effect. This study showed that in the whole trial population, the treated group lost on average 47.7 m over the 48 weeks follow up, whilst the placebo group lost on average 60.7 m. There was no significant difference between these two groups (*p* = 0.213). However, in the prespecified group with 6 MWD between 300 and 400 m, the treated group lost on average 27.0 m over the 48 weeks follow up, whilst the placebo group lost on average 69.9 m. Here the difference was significant (*p* = 0.007). A similar, post-hoc subgroup analysis was also done in the phase 3 trial of the *DMD* gene exon 51 skipping ASO Drisapersen (Goemans et al., 2018). Here, the outcome measure of interest was also the 6 MWD but there was significantly more variability in the data than was expected, leading to an overestimation of the trial power and a non-significant treatment effect. Consequently, a secondary analysis, on only those patients with a 6 MWD between 300 and 400 m who could rise from the floor, was completed. This yielded a significant treatment effect of 35.4 m (*p* = 0.039) for a 48 weeks follow up. However, this restrictive criteria approach can underestimate the treatment effect heterogeneity, due to bias sampled into the trial population. This can then cause issues for clinicians in managing patient and carer expectations of new treatments.

Matching between the treated and placebo arms is critical to allow the detection of treatment effects. Several factors can impact the treatment effect and interpretation of clinical trial results, including age, disease duration and functional status at baseline. A primary example of this is the recently announced top-line results of the Sarepta micro-dystrophin gene-therapy trial (Sarepta Therapeutics, 2021). No significant difference (*p* = 0.37) was found between the change in NSAA score for the treated and placebo arms. The investigators suggested that this may be due to a significant difference in the motor function scores at baseline between the patients aged 6 to 7, where treated patients had significantly worse scores not only on the NSAA scores, but raise time to stand from supine, 10 m walk and 100 m run at baseline compared to those on placebo (*p* = 0.005). This may have been the reason that the treated arm did not show a drug effect compared to the placebo group, as the patients who were in the treated group were more severe at baseline.

### Factors That Contribute to Disease Heterogeneity

There is significant heterogeneity of disease progression in both SMA and DMD, as well as in treatment approaches, which warrant significant consideration when designing clinical trials. It is worth noting here that the degree of variability in the patient population that is tolerable in a trial is directly related to the size of the treatment effect and the extent to which this is captured by the outcome measure.

The impact of genetic variability on this variation is not fully understood as of yet. In SMA, the number of copies of *SMN2* has been reported to correlate with disease severity but not with response to treatment (Baranello et al., 2021). However, clinical severity and treatment response vary significantly between patients with SMA, as several factors, including the interval between disease onset and initiation of therapy, and intragenic and structural variation in the *SMN* locus have been reported to possibly explain at least partly the clinical variability in SMA (Wadman et al., 2020).

In recent years, the genetic background and the differential expression of dystrophin isoforms have been proposed as key factors in the design of clinical trials (Desguerre et al., 2009). Several studies have shown that more distal mutations in the DMD gene are correlated with worse respiratory and cardiac outcomes (Yamamoto et al., 2018; Bello et al., 2020). The recent long term natural history studies in DMD patients with different genetic backgrounds have contributed to understanding motor function trajectories for DMD patients with different mutations amenable to exon skipping; those amenable to skip exons 53 and 51 display lower motor function scores at baseline and more negative changes than the other subgroups (Brogna et al., 2018; Bello et al., 2020). When it comes to recruiting populations for randomised control trials (RCTs), considerable effort should be made to ensure that the treated and placebo populations are matched in terms of genetics, along with other more standard criteria such as age and baseline motor function.

Genotype-phenotype correlation presents an additional level of difficulty in getting well matched trial populations when the trial population is further restricted. Exon skipping drugs in DMD provide an example of this, as the drugs are only appropriate for a subset of the DMD population: for example, those eligible for exon 51 skipping drugs make up ∼14% of the DMD population. It has been shown that amenability to different exon-skipping drugs correlates with disease trajectory (Brogna et al., 2018; Bello et al., 2020), and therefore it is crucial that treated and placebo patients are matched according to *DMD* genotype.

Matching on SoC can also present significant challenges in trial design. For example, in DMD GCs form much of the SoC treatments, and several studies have shown that GC use delays loss of ambulation (Bushby et al., 2004). There are, however, large amounts of variety in GC regimes, including variation in type, frequency, and dose (Griggs et al., 2013). Clinicians often prescribe specific regimes based on patient characteristics, but the true effect of each regime has not been fully understood. These differences in GC treatment add an additional level of complexity to matching in trials, especially as it is known that different GC regimes may have more significant effects on motor outcomes, such as loss of ambulation (Ricotti et al., 2013).

Beyond the issue of matching cohorts as presented above, traditional RCTs for DMD and SMA present other difficulties. The presence of a placebo arm in an RCT can be unattractive to patients and carers if the risk (typically 33% or 50%) of being randomised to the placebo arm is perceived as too high. In SMA in particular, where other disease-modifying therapies have already been approved, ethical considerations are necessary if placebo arms are included in trials. This is even truer for patients with SMA I where earlier age of treatment relative to symptom onset has been shown to result in more favourable outcomes (Finkel et al., 2017). Due to the increasing availability of disease-modifying treatments to SMA patients, future trials will need to adapt to this evolving landscape and to consider patients treated in the real world as comparators.

### Novel Trial Design With Real-World Data

The aforementioned challenges in recruiting both SMA and DMD patients into RCTs with placebo arms and sufficient duration of follow-up to detect treatment effects has led to an interest in alternative trial designs within the neuromuscular community. These alternative trial designs do not require a unique, well-matched placebo arm, but instead, aim to draw on previous data on untreated (or SoC treated) patients who can be “selected” into an augmented control arm by applying the same inclusion criteria. This is referred to as real-world evidence (RWE) by the FDA and guidance for trial design using RWE has been published recently (U.S. Department of Health and Human Services and Food and Drug Administration, 2019). There are two types of untreated populations that can serve as comparators, placebo arms from other trials and natural history studies. Both populations have their advantages and disadvantages. Historic placebo arms from completed trials are often formatted in a way that makes them easy to use as augmented controls. This is because the outcome measures for trials in a specific disease are often the same, and these outcomes are assessed at regular follow-ups with a very low proportion of missing data. However, these historic placebo arms are often fairly small, and the duration of the trials is limited, so it may become necessary to use multiple different trial placebo arms. As inclusion criteria for trials tend to be fairly restrictive, there can also be bias if the historic placebo arm (or arms) does not have sufficiently similar inclusion criteria. On the other hand, natural history data are often very large, and the trial inclusion criteria can be applied to datasets to create an augmented control arm. There are limitations with this approach, as there is an inherent bias in natural history data, as clinical decisions for patients are not randomised. Additionally, the natural history data itself can have high proportion of missing data and inaccuracies. Applying trial inclusion criteria to natural history data is not straightforward, particularly because inclusion into a new therapy trial is often a proxy measure for disease severity, with those included in trials often younger and with the most typical disease progression (Franklin and Schneeweiss, 2017). Goemans et al. (2020) has shown that change in 6 MWD, one of the most used DMD outcome measures, is consistent between six placebo arm groups and four natural history observational studies, suggesting that the choice of which set of data to use might not be so critical, and that the use of both natural history data and historic placebo arms to create augmented control arms might become more common in future trials. In keeping with current regulatory requirements, it is worth noting that if an augmented control arm is to be considered it must be specified prospectively in the trial design and should not be done retrospectively if the actual placebo arm is deemed insufficient (Committee for Medicinal Products for Human Use (CHMP), 2018b).

Lake et al. (2021) described a novel, practical method for incorporating placebo arms from other trials into the analysis of the Dystance 51 phase 2/3 trial of Suvodirsen (ClinicalTrials.gov NCT03907072), another ASO for the skipping of exon 51 of the *DMD* gene. Unfortunately, the Dystance 51 trial was stopped due to the lack of increased dystrophin expression in the open-label extension of the phase 1 Suvodirsen trial (Wave Life Sciences, 2019). This approach, using an adapted Bayesian trial design, would have been implemented for the analysis if the trial had been completed. Despite having a placebo arm itself, the design of this trial was such that the placebo arms from three historic trials (“historic placebos”) could be used to augment those randomised to placebo in the Dystance 51 trial. This augmentation was done by Bayesian meta-analysis, whereby the information from the historic placebos was dynamically “borrowed” into the augmented placebo arm. The amount of borrowing depends on how well the true placebo arm mirrored the historic placebo arms in terms of the primary endpoint. For example, if the placebos in the Dystance 51 trial act similarly to the historic placebos then more information can be borrowed into the augmented placebo arm, but if the placebo arms look different then less is borrowed. This method results in fewer patients needing to be recruited into the placebo arm to achieve the effective sample size needed in the trial.

### Clinically Relevant Endpoints

A key difficulty when designing trials for DMD and SMA is how to define an endpoint that is feasible, meaningful, and timely. In DMD for example, there are three outcomes of interest: biological, where a significant increase in dystrophin is produced in treated patients compared to untreated patients; clinical, where a significant change in clinical outcome measures are observed relative to the natural history or placebo cohorts; and patient reported, where a significant change is perceived as a clinically-relevant endpoint by patients and/or their family/carers. The FDA and EMA both have accelerated approval pathways for drugs developed to treat serious or life-threatening conditions. The FDA accepts the use of surrogate, biological trial endpoints that are considered reasonably likely to predict benefit to patients, with confirmation of clinical benefit for these drugs in the post-marketing setting as a condition of continued approval. By contrast, EMA considers that at present there are no suitable biomarkers to establish treatment efficacy and data on a clinical endpoint are required for approval.

Eteplirsen, an ASO skipping exon 51, is a crucial case study here, as it has been approved in the US but not in Europe (Committee for Medicinal Products for Human Use (CHMP), 2018b; US Food and Drug Administration, 2016). The PROMOVI Eteplirsen trial (ClinicalTrials.gov NCT02255552) found a significant difference in dystrophin production between the placebo and treated group but failed to find a significant difference in the change in motor function between the two groups. The question then arose, what is the threshold above which an increase in dystrophin levels will have a clinical effect and is it enough that a drug can be shown to work biologically but not clinically. This was one of the main reasons for the EMA CHMP rejection.

Although not included in DMD and SMA trials as the primary endpoint, patient reported outcomes (PROs) are becoming more common as secondary outcomes in recent clinical trials. For example, they are included in the Sarepta Micro-dystrophin for DMD trial (ClinicalTrials.gov NCT03769116), the Pfizer mini-dystrophin for DMD trial (ClinicalTrials.gov NCT04281485) and the ACT-DMD Ataluren trial (ClinicalTrials.gov NCT01826487). Although the most commonly used PRO measure in DMD is the Paediatric Outcomes Data Collection Instrument (PODCI) (Daltroy et al., 1998), there has been a recent drive to develop specialised PRO measures for specific disease stages for both DMD and SMA. For example, the DMD specific Quality of Life (QoL) measure, the DMD-QoL (Powell et al., 2021), and the DMD upper body specific PRO measure, the DMD Upper Limb PROM (Klingels et al., 2017), have been developed specifically for the DMD population. Additionally, both the FDA and EMA have published reports reiterating the need for patient reported outcomes for supporting claims in product labelling (Committee for Medicinal Products for Human Use (CHMP), 2005; FDA Center for Biologics Evaluation and Research, 2009).

A key issue with clinical outcome measures is ensuring that the observed change is meaningful, and we discuss here several different issues relating to that. First, we must be able to say that the observed change is real, not just variability in the measure. One part of this is knowing the minimal detectable change (MDC), for which novel methods have been developed. We must also be aware of the reliability of the score, and particularly between different centres and different assessors. The MDC is the smallest difference that indicates a real clinical change in disease. This value for 80% confidence that a change is indicative of a true change has been calculated by Muntoni et al. (2021) for the NSAA (2.78), 6 MWD (36.3), 4 stair climb (4SC) time (1.31) and 4SC velocity (0.35).

We must also take into account the inherent variability in outcome measures. This can take the form of test-retest reliability, such as in biological outcomes, where natural variation in biomarkers means that they can only be assessed with error. Additionally, inter-rater reliability can be of concern, such as in motor function assessments where there can be a discrepancy between assessors. Additionally, variation in methodology for the extraction and analysis of biomarkers can also lead to inter-rater reliability issues. Finally, the use of clinical and patient reported outcomes in populations for which the measure has not been validated can lead to significant error. All three of these factors can lead to increased noise when assessing the treatment effect.

The minimal clinically important difference (MCID) must also be considered when selecting the outcome of a clinical trial. Ideally, any reported outcome of a trial would be greater than the MCID to indicate a meaningful effect on patient quality of life. To assess true MCID patients and carers are often contacted, and their opinions assessed via questionnaires. Alternative methods to calculate the MCID include anchoring the score of interest to other scores which already have a validated MCID or calculating the MDC as a proxy for the MCID using distribution-based approaches. For example, Stolte et al., 2020 reported the MDC (as a proxy for the MCID) for the revised upper limb module (RULM) and the HFMSE between 2.9 and 6.4 and between 4.3 and 10.6 respectively. The linear NSAA is a transformed version of the NSAA ranging from 0–100, where the change in scores are standardised across the scale (Mayhew et al., 2013). The MDC (as a proxy for the MCID) for the linear NSAA in DMD was found to be 8.8 and 6.9 for boys on daily and intermittent prednisolone respectively (Mayhew et al., 2013), whilst for the 6 MWD in DMD it was found to be 28.5/31.7 (Mcdonald et al., 2013).

## Conclusion

In conclusion, translational research in neuromuscular diseases has had a pioneering role over the past decade in developing high quality, well-designed clinical trials that have led to the approval of several novel therapies. Much has been learnt on how to select patients to be recruited based on foreseen trajectories and possible responses to treatment. With the rapidly evolving technology and translation of preclinical science to patients, it will be crucial to have both robust clinical trial design and adequate treatment effect to ensure the success of future clinical trials. With many failed trials, the increasing availability of treatment to patients in the real world, and the long duration that many studies require to detect a clinical response, alternative study designs are receiving increasing consideration by industries and regulatory authorities. These include comparison with natural history cohorts and placebo arms from other trials that may become particularly relevant for rare diseases in the near future. The proper design of a clinical trial is crucial to its success in leading to the approval of the medicine products under investigation; however, the implementation of approved treatments in the real world, where there are no strict inclusion/exclusion criteria and patients’ clinical heterogeneity may be much wider, requires careful discussion and counselling with patients and caregivers to properly manage expectations. Post-marketing disease registries and databases will be essential in the coming years to collect real-world data. This in turn will help clinicians, carers, and patients alike to understand the short and long-term impact of these new neuromuscular therapies, as it has been done historically with renal and cancer registries.

## Abbreviations

6MWD, 6 minute Walking Distance; AAV, Adeno Associated Virus; ASO, Antisense Oligonucleotide; CHMP, Committee for Medicinal Products for Human Use; DMD, Duchenne Muscular Dystrophy; EMA, European Medicines Agency; FDA, US Food and Drug Administration; GC, Glucocorticoid; HFMSE, Hammersmith Functional Motor Scale Expanded; MCID, Minimal Clinically Important Difference; MDC, Minimal Detectable Change; NSAA, North Star Ambulatory Assessment; PODCI, Paediatric Outcomes Data Collection Instrument; PRO, Patient Reported Outcome; RCT, Randomised Control Trial; RULM, Revised Upper Limb Measure; RWE, Real World Evidence; SMA, Spinal Muscular Atrophy; SMN, Spinal Motor Neurone; SoC, Standard of Care.
